# Editorial: How Normal Is the New Normal? Individual and Organizational Implications of the COVID-19 Pandemic

**DOI:** 10.3389/fpsyg.2022.931236

**Published:** 2022-06-21

**Authors:** Amelia Manuti, Beatrice Van der Heijden, Peter Kruyen, Ans De Vos, Monica Zaharie, Alessandro Lo Presti

**Affiliations:** ^1^Department of Education, Psychology, Communication, Università “Aldo Moro” di Bari, Bari, Italy; ^2^Institute for Management Research, Radboud University, Nijmegen, Netherlands; ^3^Open Universiteit, Heerlen, Netherlands; ^4^Department of Marketing, Innovation, and Organisation, Ghent University, Ghent, Belgium; ^5^School of Business, Hubei University, Wuhan, China; ^6^Kingston Business School, Kingston University, London, United Kingdom; ^7^Antwerp Management School, University of Antwerp, Antwerp, Belgium; ^8^Department of Management, Babes-Bolyai University, Cluj-Napoca, Romania; ^9^Department of Psychology, Università della Campania “Luigi Vanvitelli”, Caserta, Italy

**Keywords:** HRM practices and policies, new normal, impact of the pandemic on individuals, impact of the pandemic on teams, impact of the pandemic on organizations

“How normal is the new normal?” The idea of this Research Topic started from this simple question that is tickling our imagination as scholars, employees, and—for some of us—as supervisors. The term “new normal” was coined during the 2008 financial crisis to refer to the dramatic economic, cultural, and social transformations that seriously impacting collective perceptions and individual lifestyles. During the COVID-19 pandemic in 2020, the term “new normal” reappeared to point out how the pandemic completely transformed human life, including professional identity, economic subsistence, work and family organization, children's education; and, in turn, demanding a radical revision of the traditional ways, practices and skills used to manage them.

Indeed, since the start of the pandemic, it has been evident that COVID-19 was destined to mark our history, triggering long-term effects for individuals, teams, and organizations. Although we are longing to return to our familiar routines, it is evident that everything has changed, and we still have difficulties adapting to this new normal. Accordingly, the increasing complexity of the present scenario urges us find answers for the most evident implications of the pandemic (e.g., remote working and technostress, distance management, work/life interface, economic, and job insecurity) with other eminent issues that emerged in this “new normal” phase (e.g., research on long-term effects, cross-country comparative research, how to prepare for a new health crisis, how to support workers who suffer from long-COVID, how to accommodate workers who are afraid of getting infected, how to keep the good things that the new normal has brought us, including the increased respect for health workers?).

In view of the above, the present Research Topic aims to answer some of these questions by nurturing an expert discussion on the issue, and by focusing on some emergent challenges that will most likely keep having an impact on the future workplace, conditioning workers' wellbeing and functioning, and consequently organizational performance.

In particular, the pandemic has affected both objective and subjective aspects of work experiences. It has led to the re-organization of working spaces and organizational processes, the restructuration of tasks, herewith demanding individuals to rapidly adapt to change, and having a substantial impact on the person/organization relationship (Robelski et al., 2019; Caligiuri et al., 2020; Carnevale and Hatak, 2020). Connected to these changes in working spaces and organizational processes, different stakeholders in organizations (e.g., employees, supervisors, and top management) are experiencing several transformations related to new forms of distance management and performance control. Issues like motivation, coaching and mentoring, organizational support, conflict management, and employee development are more important than ever for organizational survival. At the same time, there is still a limited understanding of how objective and subjective aspects of employees' working experiences have been affected by the changes due to COVID-19, let alone what organizations can do to safeguard employee wellbeing and functioning.

We argue that Human Resource Management (HRM) plays a crucial role in helping all parties involved to cope with the enormous challenges posed by the changes triggered by the pandemic. More specifically, HRM professionals should function as key strategic partners, and focus on developing a new culture of change that can inspire workers to adjust to the new normal (Gould-Williams, 2007; Demo et al., 2012; Manuti et al., 2020). As such, HRM professionals are indispensable in the light of protecting all workers' career sustainability (i.e., happiness, health, and productivity) over time (De Vos et al., 2020).

Most contributions in this Research Topic underline the central role played by management in supporting employees to deal with the effects of the pandemic both in their private and professional life. Supervisors are key figures who can buffer the effects of some negative organizational actions. For instance, the study by Spagnoli et al. highlights that for remote workers a low authoritarian leadership style has a moderating effect on the relationship between workaholism and technostress. The qualitative investigation by Ripamonti et al. underlines managers' responsibility in constructing a positive environment (an HRM ethics of care as the authors write) by adopting a people-based approach wherein employees are supported, trust and engagement are created, and the quality of the relationships within the organization is cherished, especially during times of great change and uncertainty like the one drawn by the pandemic. In a similar vein, Coun et al. make an important contribution by showing the positive relationship between empowering leadership style and employees' innovative work behavior, even in intense remote work contexts. In line with these empirical findings, the theoretical paper by Chen poses an important question analyzing the managerial point of view in dealing with the new normal: How can HR practitioners enhance the role of culture in the new work model, given that they could be important promoters of corporate culture? The author offers a series of reflections on the psychological impact of “working from home” (WFH) on workers wellbeing and on their performance, and addresses what is in his view one of the most urgent challenges for HRM practitioners in this scenario: the need to reformulate traditional training approaches and to develop innovative models that could equip workers with the skills needed to cope with new job demands, in order to reduce stress and work/life conflict.

Parallel to these studies that have mostly focused on the organizational perspective, other studies encompassed in this Research Topic consider the individual's point of view in dealing with the ongoing changes. Adopting the Job Demands-Resources model, these studies show how the pandemic has exacerbated the negative perceptions of some specific job demands (e.g., workload and social isolation), that because of remote working (Pulido-Martos et al.) profoundly affected the quality of life of workers (Barbieri et al.), resulted in behavioral stress (Ingusci et al.), and impacted the work/life interface (De Simone et al.), job insecurity (De Angelis et al.; Vieira dos Santos et al.), and financial insecurity (Rasdi et al.) have proven to be the most diffused psychological consequences of the pandemic, together with a lower work engagement (Reinwald et al.), somatization and distress (Franck et al.), and poor wellbeing (Rus et al.), especially for healthcare professionals who were among the most challenged category of workers. By adopting an individual perspective, from the scholarly work in our Research Topic, we conclude that fostering job crafting behaviors, that is providing workers with opportunities to rely upon organizational job resources (e.g., organizational, and social support) as well as on their personal resources (self-efficacy, commitment to organizational change, vigor at work), could help workers' attitudes and behaviors in the new normal.

To conclude, the rich scholarly work that is presented in this Research Topic offers several lessons for individuals and organizations for a positive transition to the new normal in the post-pandemic scenario. Yet, as argued earlier and clearly shown by the studies presented above, the huge and radical transformations that have impacted the working context have reshaped not simply the objective conditions of work but also the subjective experiences of work. Specifically, beliefs, attitudes, feelings, and practices traditionally linked to one's own professional experience and to the organizational identity have been reformulated. As a matter of fact, organizations, being social systems, need to carefully consider this evidence and to rethink their practices and policies accordingly, to protect and further enhance all workers' health, happiness, and productivity over time, whether in times of crises or not.

## Author Contributions

All authors listed have made a substantial, direct, and intellectual contribution to the work and approved it for publication.

## Conflict of Interest

The authors declare that the research was conducted in the absence of any commercial or financial relationships that could be construed as a potential conflict of interest.

## Publisher's Note

All claims expressed in this article are solely those of the authors and do not necessarily represent those of their affiliated organizations, or those of the publisher, the editors and the reviewers. Any product that may be evaluated in this article, or claim that may be made by its manufacturer, is not guaranteed or endorsed by the publisher.
